# Nuclear HDAC6 inhibits invasion by suppressing NF-κB/MMP2 and is inversely correlated with metastasis of non-small cell lung cancer

**DOI:** 10.18632/oncotarget.4749

**Published:** 2015-09-15

**Authors:** Chih-Jen Yang, Yu-Peng Liu, Hong-Ying Dai, Yow-Ling Shiue, Chia-Jung Tsai, Ming-Shyan Huang, Yao-Tsung Yeh

**Affiliations:** ^1^ Graduate Institute of Medicine, College of Medicine, Kaohsiung Medical University, Kaohsiung, Taiwan; ^2^ Department of Internal Medicine, Kaohsiung Municipal Ta-Tung Hospital, Kaohsiung Medical University Hospital, Kaohsiung Medical University, Kaohsiung, Taiwan; ^3^ Division of Pulmonary and Critical Care Medicine, Department of Internal Medicine, Kaohsiung Medical University, Kaohsiung, Taiwan; ^4^ Cancer Center, Kaohsiung Medical University Hospital, Kaohsiung, Taiwan; ^5^ Department of Genome Medicine, Kaohsiung Medical University, Kaohsiung, Taiwan; ^6^ Center for Infectious Disease and Cancer Research, Kaohsiung Medical University, Kaohsiung, Taiwan; ^7^ Department of Medical Laboratory Sciences and Biotechnology, Fooyin University, Kaohsiung, Taiwan; ^8^ Institute of Biomedical Science, National Sun Yat-Sen University, Kaohsiung, Taiwan

**Keywords:** HDAC6, NF-κB, MMP2, metastasis, lung cancer

## Abstract

Histone deacetylase 6 (HDAC6) is a unique member of the histone deacetylase family. Although HDAC6 is mainly localized in the cytoplasm, it can regulate the activities of the transcription factors in the nucleus. However, a correlation of intracellular distribution of HDAC6 with tumor progression is lacking. In this study, we found that a low frequency of nuclear HDAC6-positive cells in tumors was associated with distant metastasis and a worse overall survival in 134 patients with non-small cell lung cancer (NSCLC). Ectopic expression of wild-type HDAC6 promoted migration and invasion of A549 and H661 cells. However, the enforced expression of nuclear export signal-deleted HDAC6 inhibited the invasion but not the migration of both cell lines. The inhibitory effect of nuclear HDAC6 on invasion was mediated by the deacetylation of the p65 subunit of nuclear factor-κB, which decreased its DNA-binding activity to the *MMP2* promoter, leading to the downregulation of MMP2 expression. Our findings indicated that the loss of nuclear HDAC6 may be a potential biomarker for predicting metastasis in patients with NSCLC.

## INTRODUCTION

Histone deacetylases (HDACs) regulate the post-translational modification of lysine residues in histone tails by deacetylation, thereby controlling gene expression. HDACs comprise four classes on the basis of their primary homology to the yeast histone deacetylases [1]. HDAC6, which belongs to class IIb, is a unique isoform among HDACs because of its two homologous tandem catalytic domains (all other HDACs have only one) [2, 3]. HDAC6 is predominately expressed in the cytoplasm because its amino acid sequence contains a nuclear export signal (NES) and Ser-Glu-containing tetrapeptide (SE14) [4].

HDAC6 may perform different functions and possess different activities in different cancers [5]. In some human cancers, such as ovarian cancer, HDAC6 is linked to the oncogenic transformation [6]. Aside from solid tumors, HDAC6 is consistently upregulated in primary acute myeloid leukemia blasts and some myeloblasts, implying an oncogenic role for HDAC6 [7]. In breast cancer, HDAC6 mRNA expression is more prevalent in less advanced, less aggressive tumors, and is associated with better prognosis [8, 9]. However, in another study, HDAC6 protein levels were not associated with the prognosis of patients with breast cancer, although HDAC6 may be a prognostic indicator for patients with estrogen receptor-positive breast cancer [9]. The role of HDAC6 in hepatocellular carcinoma is controversial [10, 11], and its association with prognosis in lung cancer is still unknown.

Several cytoplasmic and nuclear substrates and interacting partners of HDAC6 have been identified. Cytoplasmic HDAC6 has been reported to interact with α-tubulin, cortactin, heat shock protein 90, β-catenin, and peroxiredoxins and to survive to regulate tumor growth, apoptosis, and migration. Although HDAC6 is normally localized in the cytoplasm, a fraction of HDAC6 enters the nucleus in response to various stimuli [12, 13]. It has been demonstrated that HDAC6 expression levels may not be as critical as its sub-cellular localization, providing a potential explanation for previous clinical discrepancies [14]. The functional role of nuclear HDAC6 is largely unknown. HDAC6 has been reported to directly bind to the nuclear factor-κB (NF-κB) in the nucleus and to inhibit the transcription of the *H^+^*-K*^+^ ATPase α2 subunit* gene in the mouse inner medullary collecting duct 3 cells [15]. However, it is unclear how HDAC6 regulates the transcriptional activity and functional effect of NF-κB in cancer cells.

NF-κB controls over 400 genes that are involved in normal physiology and pathological conditions, such as inflammation and cancer [16, 17]. NF-κB signaling can be activated by classical and nonclassical pathways. NF-κB functions only as a dimer; The homo- or heterodimers of p50 and p52 subunits of NF-κB have been reported to repress transcription, and the p50/RelA (p65) is active for DNA binding [18]. Activation of NF-κB relies on the phosphorylation of p65 in the cytoplasm or in the nucleus. These processes are stimulus and/or cell-type specific. Recently, other modifications of NF-κB, including acetylation, nitrosylation, ubiquitination, neddylation, and sumoylation, have also been reported to play important roles in regulating NF-κB function [19]. Acetylation of the p65 subunit on lysine residues 218, 221, and 310 increases its DNA-binding ability [20]. In contrast, deacetylation of p65 at Lys122 and Lys123 by HDAC3 has been demonstrated to reduce the DNA-binding affinity of NF-κB [21].

In present study, we demonstrated that the nuclear translocation frequency of HDAC6 is associated with distant metastasis and overall survival in patients with non-small cell lung cancer (NSCLC). Nuclear HDAC6 directly bound to NF-κB, leading to the deacetylation of NF-κB and the downregulation of matrix metalloproteinase 2 (MMP2). This downregulation inhibits the invasion of lung cancer cells.

## RESULTS

### HDAC6 expression levels are not correlated with NSCLC prognosis

**Figure 1:**
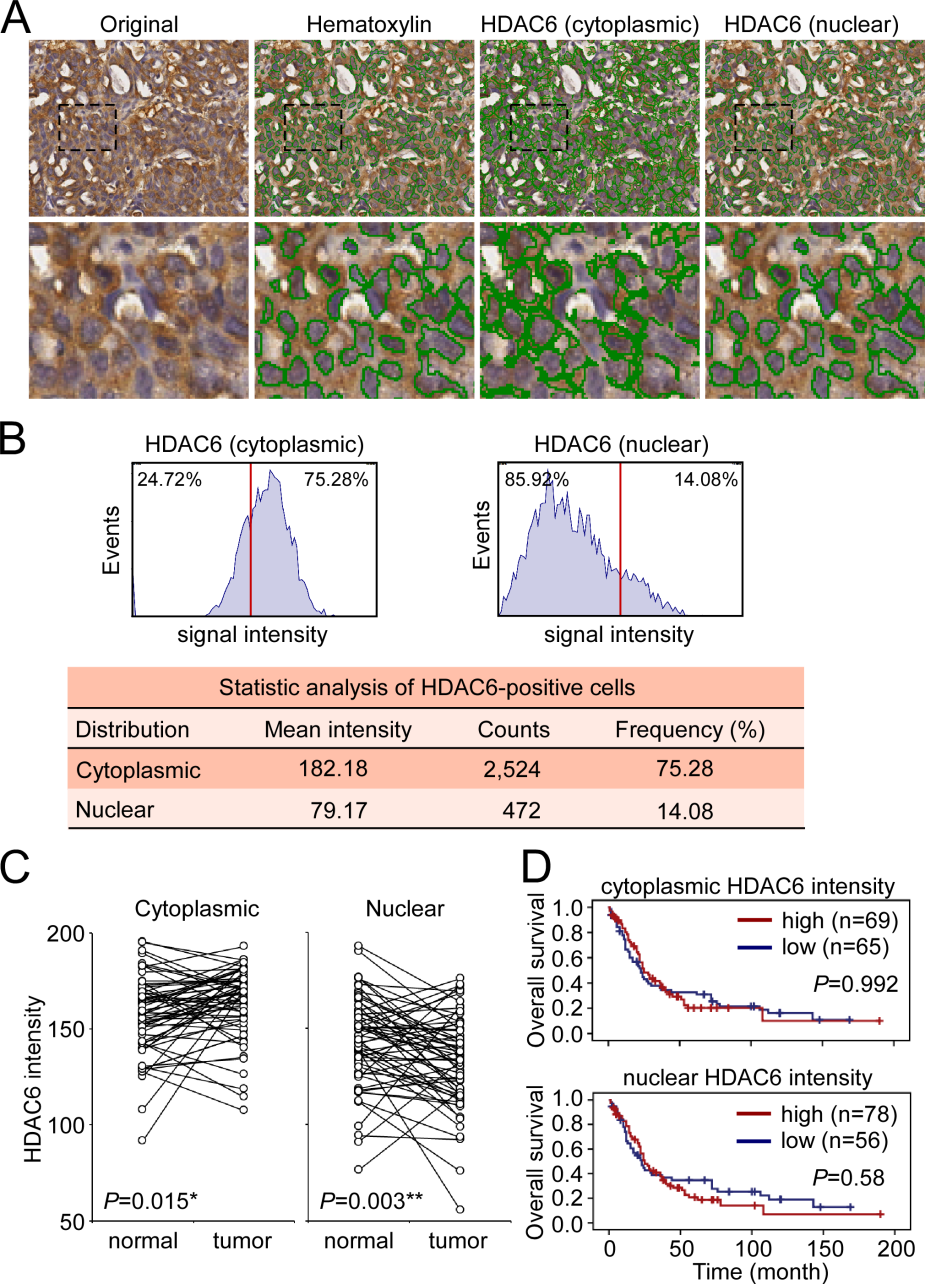
Analysis of HDAC6 cytoplasmic and nuclear localization **A.** Representative image showed the identification of hematoxylin-stained nucli and cytoplasmic and nuclear HDAC6 in a section of paraffin-embedded NSCLC specimen using HistoQuest software. **B.** Quantitative analysis of the intensity and frequency of cytoplasmic and nuclear HDAC6. Red line indicated the 50% cutoff of the HDAC6 intensity. **C.** The signal intensity of cytoplasmic and nuclear HDAC6 in the paired normal tissues and tumors in 63 NSCLC patients was plotted. *P < 0.05, **P < 0.01. **D.** Kaplan-Meier log-rank analysis for the overall survival of the 134 NSCLC patients with low and high intensity of cytoplasmic and nuclear HDAC6 in the tumor regions.

We used immunohistochemistry (IHC) to investigate the expression and sub-cellular localization of HDAC6 protein in a tissue array that comprised clinical specimens from 134 patients with NSCLC. The 134 patients ranged in age from 31 to 82 years (mean, 61.6 years; SD, 10.4 years) and comprised 63 males and 71 females. The clinicopathological features of the patients are shown in Supplementary Table 1. The IHC results revealed that cells with cytoplasmic and/or nuclear HDAC6 immunoreactivity could be observed in all specimens. We further quantified the intensity of cytoplasmic and nuclear HDAC6 staining signals (range from 0 to 255) using the HistoQuest system, which allows quantification of the cell number and the HDAC6 immunoreactivity in the cytoplasm and nucleus of each cell (Figure 1A). On an average, 2518 ± 963 cells were counted in the tumor regions of each section. Quantification of the cytoplasmic and nuclear HDAC6 intensities of each sample was plotted (Figure 1B). We examined the cytoplasmic and nuclear HDAC6 intensity in 63 sets of matched samples from primary lung tumors and normal adjacent tissues (N-T paired). In 41 of the 63 patients, the cytoplasmic HDAC6 levels were significantly upregulated in tumors compared with its expression in normal tissues (*P* = 0.015). In contrast, in 43 of the 63 patients, the nuclear HDAC6 levels were significantly downregulated in the tumors compared with its expression in normal tissues (*P* = 0.003) (Figure 1C). To evaluate the prognostic significance of the cytoplasmic and nuclear HDAC6 protein levels, we scored the HDAC6 intensity in tumor samples from the 134 patients with NSCLC on a scale from zero (low expression) to three (high expression) on the basis of the staining intensity in the cytoplasm and nucleus (Supplementary Figure 1). In a Kaplan–Meier log rank analysis, the HDAC6 staining intensity (low: scores 0 and 1; high: scores 2 and 3) in neither the cytoplasm nor the nucleus was statistically correlated with the overall or disease-free survival of patients with NSCLC (Figure 1D).

### Frequency of HDAC6 nuclear localization is significantly correlated with metastasis and poor prognosis in patients with NSCLC

**Figure 2:**
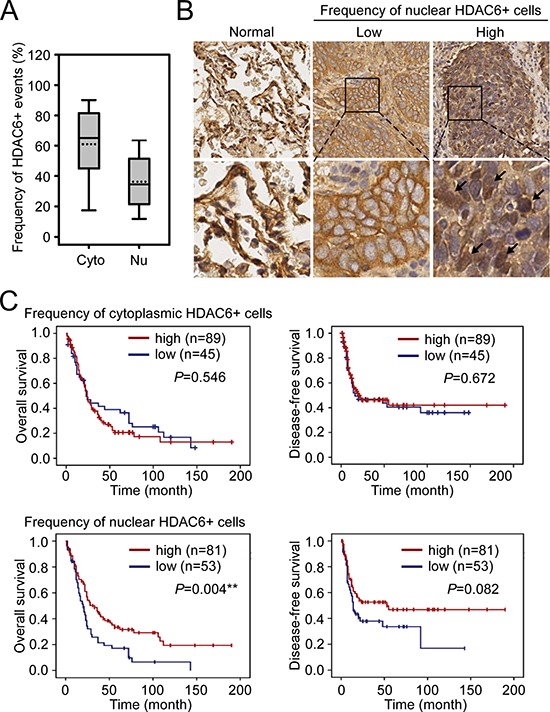
The association of nuclear HDAC6 frequency with NSCLC patient survival **A.** The frequency (%) of cytoplasmic and nuclear HDAC6 in the tumor parts of the 134 NSCLC patients. Cyto, cytoplasmic HDAC6; Nu, nuclear HDAC6. **B.** The representative images demonstrated the low and high frequency of nuclear HDAC6+ cells in tumor regions. Images of HDAC6 staining in two normal tissues were shown for comparison. **C.** Kaplan-Meier log-rank analysis for the overall and disease-free survival of the 134 NSCLC patients with low and high frequency of cytoplasmic (upper panels) and nuclear (lower panels) HDAC6 in the tumor regions. **P < 0.01.

We further analyzed the frequency of cytoplasmic and nuclear HDAC6 expression in the tumor samples. From the representative sample provided in Figure 1A and 1B, among the 2,996 tumor cells in the section, 75.28% and 14.08% of the cells were positive for cytoplasmic and nuclear HDAC6 localization, respectively (Figure 1B). The frequency distribution of the cytoplasmic and nuclear HDAC6-positve cells in the tumor sections from the 134 patients is shown in Figure 2A. Over 70% of patients had tumors demonstrating >50% cytoplasmic HDAC6-positive cells, whereas approximately 70% of patients had tumors with >20% nuclear HDAC6-positive cells (Supplementary Table 2). To evaluate the prognostic value of the cytoplasmic and nuclear localization of HDAC6, we divided the patients into those with a low frequency and those with a high frequency of HDAC6-positive cells using the cutoff values of 50% and 20% for cytoplasmic and nuclear localization, respectively (Figure 2B). A Kaplan–Meier analysis revealed that a low frequency of HDAC6 nuclear localization was significantly correlated with worse overall survival in the patients with NSCLC (*P* = 0.004) and had a trend toward worse disease-free survival (*P* = 0.082). The frequency of HDAC6 cytoplasmic localization was not correlated with the survival of patients (*P* = 0.546 and *P* = 0.672 for overall survival and disease-free survival, respectively, Figure 2C). Furthermore, chi-square analysis between the frequency of HDAC6 nuclear localization and clinicopathological characteristics revealed that the low frequency of HDAC6 nuclear localization was significantly associated with distal metastasis in patients (*P* = 0.031) as well as disease recurrence (*P* = 0.019; Table 1), whereas the frequency of cytoplasmic HDAC6 was not correlated with any clinicopathological feature in these patients (Supplementary Table 3). Univariate and multivariate Cox regression analysis demonstrated that the frequency of HDAC6 nuclear localization, lymph node involvement, and distal metastasis were found to be significant predictors of patient outcome (Table 2). In contrast, the HDAC6 cytoplasmic and nuclear intensities and the HDAC6 cytoplasmic frequency had no significant prediction power for patient survival. Together, these findings indicate that the reduction of HDAC6 nuclear localization frequency in tumors is highly correlated with metastasis and poor prognosis in patients with NSCLC.

**Table 1:** Relationship between the frequency of HDAC6 nuclear localization and clinicopathological factors in 134 NSCLC patients

Nuclear HADC6+ frequency			
Characteristics	Low (*n* = 53)	High (*n* = 81)	*P value
Age			0.434^†^
Years (mean ± SD)	60.7 ± 10.8	62.2 ± 10.1	
Gender			0.215^‡^
Male	32	39	
Female	21	42	
Smoking status			
No	24	54	
Yes	29	27	
Histological type			0.517^‡^
Adenocarcinoma	29	52	
Squamous cell carcinoma	19	24	
Large cell carcinoma	5		
Stage			0.400^‡^
I	7	11	
II	11	25	
III	13	22	
IV	22	23	
Tumor status			0.077^‡^
T1	9	13	
T2	21	49	
T3	4	4	
T4	19	15	
Lymph node status			0.935^‡^
N0	23	30	
N1–3	33	51	
Distal metastasis status			0.031^‡^*
M0	29	59	
M1	24	22	
Recurrence status			0.097^‡^
No	13	36	
Yes	40	45	

* *P* value < 0.05 was considered statistically significant (Student's *t* test for continuous variables and Pearson chi-square test for categorical variables). SD represents standard deviation. The tumor stage, tumor, lymph node, and distant metastasis status were classified according to the international system for staging lung cancer.

† Student's test

‡ Chi-square test

**Table 2:** Cox univariate and multivariate regression analysis of prognostic factors for overall (OS) and disease-free (DFS) survival in 134 lung cancer patients

Cox univariate analysis (OS)			
Variables	Comparison	HR (95% CI)	*P* value
T	T1-T2; T3-T4	1.904 (1.252–2.895)	0.003*
N	N0; N1-N3	2.327 (1.555–3.481)	<0.001*
M	M0; M1	2.642 (1.742–4.007)	<0.001*
Cytoplasmic HDAC6 intensity	Low; High	0.998 (0.668–1.492)	0.993
Nuclear HDAC6 intensity	Low; High	1.121 (0.744–1.688)	0.585
Cytoplasmic HDAC6 frequency	Low; High	1.173 (0.744–1.738)	0.552
Nuclear HDAC6 frequency	Low; High	0.565 (0.378–0.843)	0.005**

Cox multivariate analysis (OS)			
Variables	Comparison	HR (95% CI)	*P* value
T	T1-T2; T3-T4	1.195(0.752–1.900)	0.450
N	N0; N1-N3	2.159(1.394–3.344)	0.001**
M	M0; M1	1.965 (1.244–3.105)	0.004**
Cytoplasmic HDAC6 intensity	Low; High	1.130 (0.604–2.114)	0.703
Nuclear HDAC6 intensity	Low; High	1.520 (0.852–2.713)	0.156
Cytoplasmic HDAC6 frequency	Low; High	1.260 (0.666–2.385)	0.477
Nuclear HDAC6 frequency	Low; High	0.479 (0.295–0.780)	0.003**

Cox univariate analysis (DFS)			
Variables	Comparison	HR (95% CI)	*P* value
T	T1-T2; T3-T4	1.892 (1.135–3.153)	0.014
N	N0; N1-N3	1.493 (1.220–1.826)	<0.001*
M	M0; M1	2.420 (1.460–4.009)	0.001*
Cytoplasmic HDAC6 intensity	Low; High	1.025 (0.626–1.678)	0.921
Nuclear HDAC6 intensity	Low; High	1.341 (0.806–2.232)	0.258
Cytoplasmic HDAC6 frequency	Low; High	0.897 (0.539–1.494)	0.677
Nuclear HDAC6 frequency	Low; High	0.653 (0.399–1.069)	0.093

Cox multivariate analysis (DFS)			
Variables	Comparison	HR (95% CI)	*P* value
T	T1-T2; T3-T4	1.313 (0.737–2.342)	0.356
N	N0; N1-N3	2.096 (1.227–3.579)	0.007**
M	M0; M1	1.972 (1.108–3.512)	0.021*
Cytoplasmic HDAC6 intensity	Low; High	1.409 (0.591–3.360)	0.440
Nuclear HDAC6 intensity	Low; High	2.394 (1.162–4.933)	0.018*
Cytoplasmic HDAC6 frequency	Low; High	0.628 (0.275–1.431)	0.268
Nuclear HDAC6 frequency	Low; High	0.547 (0.300–0.996)	0.049*

* *P* value <0.05 and

** *P* value <0.01 were considered statistically significant.

### Ectopic expression of nuclear *HDAC6* decreases invasion ability of lung cancer cells

**Figure 3:**
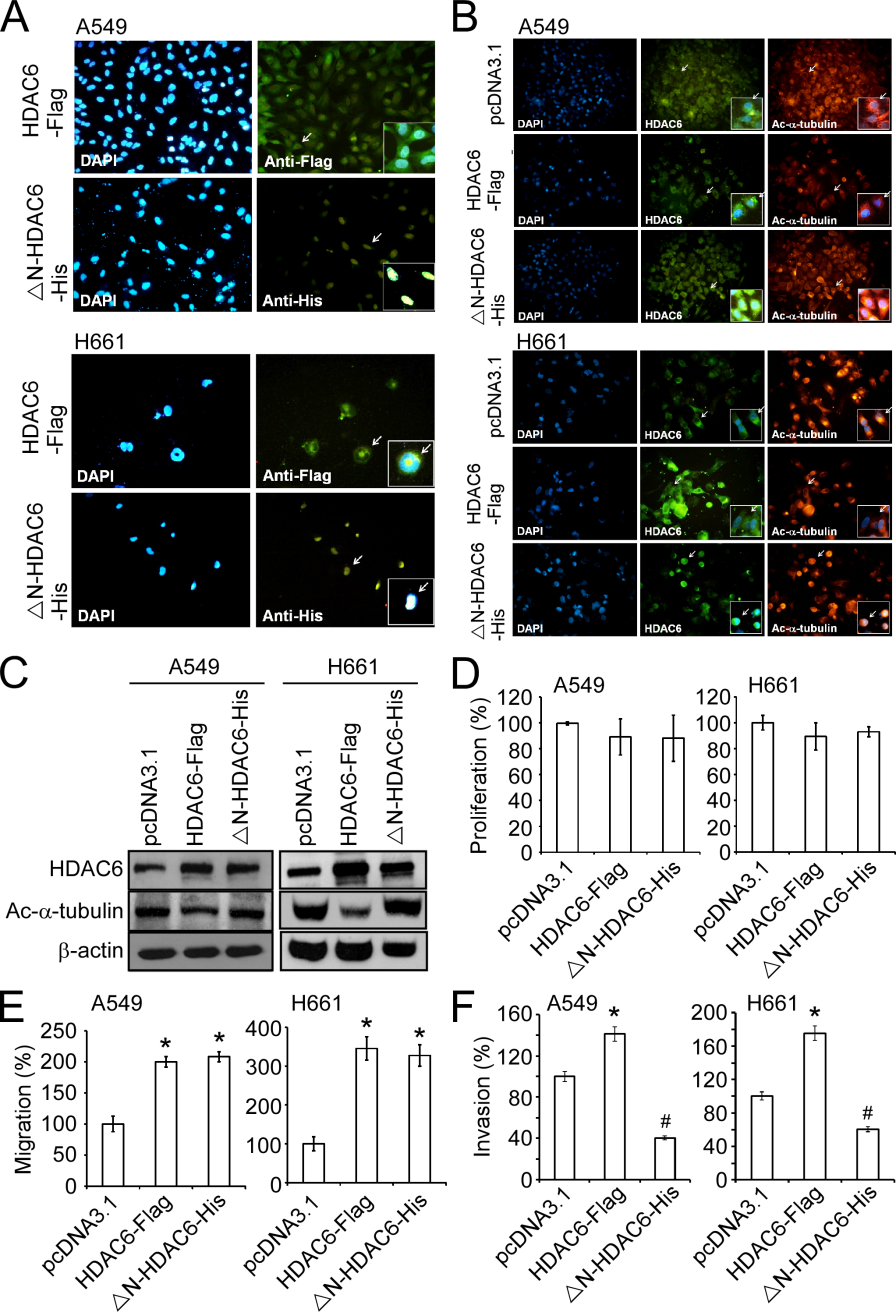
Nuclear expression of HDAC6 inhibited invasion of NSCLC cell lines **A.** Immunofluorescence images showed the intracellular localization of HDAC6-Flag and ΔN-HDAC6-His using the antibodies against Flag and His tag (green) in A549 (upper panels) and H661 (lower panels) cell lines. The nucli were stained by DAPI (blue). Arrows indicated the cells shown in the inserts with higher magnification. **B.** Immunofluorescence images showed the intracellular localization of HDAC6-Flag or ΔN-HDAC6-His (middle panels; green) and acetylated α-tubulin (right panels; red) in A549 (upper panels) and H661 (lower panels) cell lines. The nucli were stained by DAPI (left panels; blue). Arrows indicated the cells shown in the inserts with higher magnification. **C.** A549 and H661 cells were transfected with pcDNA3.1, HDAC6-Flag or ΔN-HDAC6-His, and the expression of HDAC6, acetylated α-tubulin and β-actin were analyzed by Western blot. **D.** The cell proliferation of pcDNA3.1-, HDAC6-Flag- or ΔN-HDAC6-His-transfected A549 and H661 cells were analyzed by XTT assay. **E.** The migration of pcDNA3.1-, HDAC6-Flag- or ΔN-HDAC6-His-transfected A549 and H661 cells were analyzed by wound-healing assay. *P < 0.05, compared with the pcDNA3.1 control. **F.** The invasion of pcDNA3.1-, HDAC6-Flag- or ΔN-HDAC6-His-transfected A549 (left) and H661 (right) cells were analyzed by transwell invasion assay. *P < 0.05, up-regulation, compared with the pcDNA3.1 control. #*P* < 0.05, down-regulation, compared with the pcDNA3.1 control.

To reveal the functional role of nuclear HDAC6 on the mobility of lung cancer cells, expression plasmids encoding a flag-tagged, wild-type HDAC6 (HDAC6-Flag) and a his-tagged, nuclear export signal (NES)-deleted HDAC6 (ΔN-HDAC6-His) were constructed. Immunofluorescence and Western blotting demonstrated that the ectopic HDAC6-Flag was localized to both the cytoplasm and nucleus, and the ΔN-HDAC6-His was primarily expressed in the nucleus (Figure 3A and Supplementary Figure 2). It has been revealed that HDAC6 contributes to the deacetylation of α-tubulin and enhances cell mobility [22]. Indeed, the results from IHC and Western blotting revealed that ectopic expression of HDAC6-Flag reduced the acetylation of α-tubulin in A549 and H661 cells; however, ΔN-HDAC6-His had no effect on α-tubulin modification (Figure 3B and 3C). We found that both HDAC6-Flag and ΔN-HDAC6-His facilitated cell migration, which was evaluated in a wound-healing assay (Figure 3D). This result suggests that nuclear HDAC6 may potentiate cell migration through mechanisms other than the inhibition of α-tubulin acetylation. We next investigated the effect of nuclear HDAC6 on invasiveness using a Matrigel-coated transwell assay. These results reported that ΔN-HDAC6-His significantly inhibited the invasiveness of A549 and H661 cells, whereas HDAC6-Flag promoted their invasiveness. (Figure 3E).

### Ectopic expression of ΔN-HDAC6-His reduces MMP2 expression

**Figure 4:**
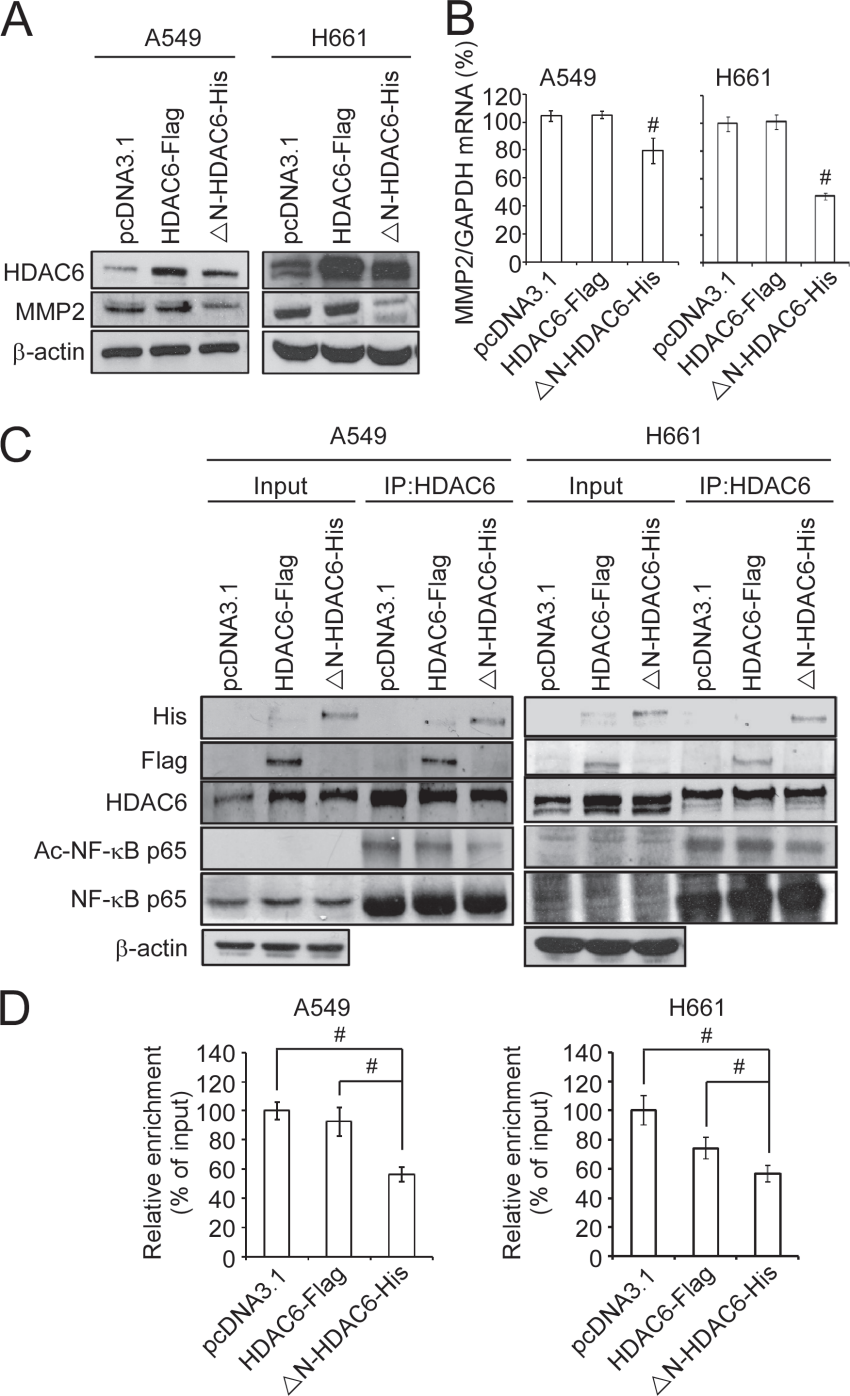
Nuclear HDAC6 inhibits MMP2 through de-acetylating NF-κB p65 subunit **A.** A549 and H661 cells were transfected with pcDNA3.1, HDAC6-Flag or ΔN-HDAC6-His, and the expression of HDAC6, MMP2 and β-actin were analyzed by Western blot. **B.** The mRNA expression of MMP2 was analyzed by real-time PCR. **C.** The interaction of HDAC6 and NF-κB was analyzed by immunopreciptation using the antibody against HDAC6 in pcDNA3.1-, HDAC6-Flag- or ΔN-HDAC6-His-transfected A549 (left) and H661 (right) cells. **D.** Chromatin immunoprecipitation was performed to the pcDNA3.1-, HDAC6-Flag- or ΔN-HDAC6-His-transfected A549 (left) and H661 (right) cells. Real-time PCR was performed using the primers for NF-κB binding sites in *MMP2* promoter.

It has been demonstrated that the enhancement of lung cancer cell invasion can be mediated through the NF-κB-MMP2 axis. Therefore, we hypothesized that the reduced cell invasiveness caused by nuclear HDAC6 may result from the inhibition of MMP2 expression. Indeed, the knockdown of MMP2 by shRNAs significantly inhibited the invasiveness of A549 and H661 cells (Supplementary Figure 3). The results of Western blotting demonstrated that the ectopic expression of ΔN-HDAC6-His significantly downregulated MMP2 protein expression in A549 and H661 cells, whereas HDAC6-Flag slightly increased MMP2 protein expression compared with the vector-only control (Figure 4A). In parallel, the results from real-time PCR demonstrated that ectopic expression of ΔN-HDAC6-His decreased MMP2 mRNA expression levels, suggesting that nuclear HDAC6 is able to transcriptionally regulate MMP2 expression in lung cancer cells. (Figure 4B).

A previous study has demonstrated that acetylation of the NF-κB p65 subunit at Lys218, Lys221, and Lys310 enhances its transactivation and DNA-binding activity that promotes the expression of downstream target genes [20]. Because NF-κB is one of the major regulators of *MMP2* transcription and is associated with metastasis [23], we were interested to understand whether nuclear HDAC6 interacts with NF-κB. To examine the interaction of HDAC6 with NF-κB, H661 cells were transfected with the plasmids pcDNA3.1, HDAC6-Flag, or ΔN-HDAC6-His, and then the transfected cells were subjected to immunoprecipitation using HDAC6 antibody. The results demonstrated that endogenous HDAC6 could bind to the NF-κB p65 subunit (Figure 4C). In addition, ectopic expression of ΔN-HDAC6-His significantly decreased the acetylation of NF-κB p65 subunit (Figure 4C). This deacetylation of the NF-κB p65 subunit occurred in the nucleus (Supplementary Figure 4). These results suggest that the ΔN-HDAC6-His-induced MMP2 downregulation may be because of the deacetylation of the NF-κB p65 subunit. Next, chromatin immunoprecipitation was performed to examine the DNA-binding activity of NF-κB on MMP2 promoter in pcDNA3.1-, HDAC6-Flag-, and ΔN-HDAC6-His-transfected H661 cells. As shown in Figure 4D, the DNA-binding activity of NF-κB was significantly reduced in ΔN-HDAC6-His-transfected H661 cells. These results indicate that nuclear HDAC6 may induce the deacetylation of NF-κB, leading to a reduction in its DNA-binding activity and MMP2 downregulation, which inhibits the invasiveness of NSCLC cells.

## DISCUSSION

In this study, we analyzed the intensity and frequency of cytoplasmic and nuclear HDAC6 in clinical specimens from 134 patients with NSCLC. A low frequency of nuclear HDAC6-positive cells significantly correlated with distant metastasis and poor overall survival in patients with NSCLC. In our *in vitro* study, we found that the ectopic expression of nuclear HDAC6 reduced the invasiveness of lung cancer cells, which may result from HDAC6-mediated deacetylation of NF-κB, which downregulated MMP2 expression. Our results indicate that the nuclear translocation of HDAC6 functionally inhibits the invasiveness of lung cancer cells that may occur through the inhibition of MMP2 expression.

Previous studies from other groups provided *in vitro* evidence demonstrating that HDAC6 may be a potential therapeutic target for platinum-refractory NSCLC [24, 25]. However, in our study, IHC of clinical specimens revealed that the increase in HDCA6 nuclear translocation reduced the risk of metastasis in NSCLC. The intensity and frequency of cytoplasmic HDAC6 were not associated with the clinicopathological characteristics or survival of patients with NSCLC. Therefore, our data indicate that the elevated frequency of nuclear HDAC6 is a significant predictor for better patient survival because of its inverse correlation with metastasis that in turn, may be because of the inhibition of MMP2 expression.

In our study, ectopic expression of HDAC6 and ΔN-HDAC6-His, which restricted HDAC6 to the nucleus, enhanced the migration ability of lung cancer cells. Although cytoplasmic HDAC6 has been demonstrated to promote cell migration by modulating microtubule acetylation [26], several lines of evidence suggest that HDAC6 can also reside in the nucleus and interact with nuclear proteins, such as HDAC11, sumoylated p300, LCoR, NF-κB, and Runx2 [27, 28]. HDAC6 binds to RUNX2 in the nucleus and suppresses the transcription of downstream genes, such as *p21-* and p53-targeting genes [28, 29]. The transcriptional activity of RUNX2 has been linked to the migration and invasion in different cancer types [30, 31]. Thus, we speculate that the nuclear HDAC6 may interact with its binding partners and regulate the transcription of downstream target genes, which are involved in the regulation of cell migration. Further experiments are required to explore these migration-associated genes and the regulatory mechanisms influenced by nuclear HDAC6.

Increased MMP2 expression predicts tumor recurrence and unfavorable outcomes in NSCLC [32]. MMP2 is a more sensitive predictor of lung cancer progression, metastasis, and survival than MMP9 [33]. In this study, nuclear HDAC6 could interact with and deacetylate NF-κB to reduce the DNA-binding activity of NF-κB on the MMP2 promoter in NSCLC cells, which reduced the expressions of MMP2 transcript and protein. MMP2 and MMP9 expression via the activation of NF-κB signaling promotes cancer cell migration and invasiveness [34]. In addition, MMP2 downregulation through the NF-κB-dependent pathway has been documented in cancer cells [35]. Here we presented a novel mechanism of NF-κB-mediated MMP2 regulation nuclear HDAC6 may serve as a transcriptional repressor attenuating MMP2 expression via deacetylation of NF-κB in NSCLC cells. Deacetylation of NF-κB is closely related to its DNA binding activity and the activation of downstream genes [36, 37].

A previous study has reported that acetylation of the NF-κB p65 subunit at Lys122 and Lys123 reduces its DNA binding activity and inhibits the expression of downstream target genes [21]. However, acetylation of p65 at Lys218, Lys221, and Lys310 enhances the nuclear translocation and DNA-binding activity of NF-κB [20]. Thus, we postulate that HDAC6 binding may result in the deacetylation of NF-κB at specific amino acid residues, such as Lys218, Lys221, and Lys310, to inactivate the transcriptional activity of NF-κB. However, further experiments, such as site-directed mutagenesis, to create point mutations at these lysine residues of p65 are required to test this hypothesis.

The sub-cellular localization of HDAC6, instead of its overall expression, in NSCLC has been reported, for the first time, to be a predictor of patient survival. In this study, we provided evidence demonstrating that increased nuclear translocation of HDAC6 prevented invasion by lung cancer cells. The regulatory mechanism responsible for HDAC6 nuclear translocation is still unclear. A recent study demonstrated that apolipoprotein E4 (ApoE4) increased the nuclear translocation of HDAC6 and inhibited the expression of brain-derived neurotrophic factor through low-density lipoprotein receptor-related protein 1 receptor-mediated signaling in neurons [38]. In contrast, activation of protein kinase C ε (PKCε) reversed ApoE4-induced nuclear translocation of HDAC6. Moreover, acetylation of the lysine residues in the N-terminal nuclear localization signal region of HDAC6 protein by p300 blocks the interaction of HDAC6 with the nuclear import protein importin-α and reduces its nuclear translocation [27]. Because HDACs play significant roles in numerous biological processes and diseases, particularly in cancers, many HDAC inhibitors have been discovered and used in several clinical trials. However, HDACs are also responsible for maintaining the physiological functions of normal cells, suggesting the predictable side effects of HDAC inhibitors. Our study demonstrated that an increase in the nuclear translocation of HDAC6 prevented the invasion of lung cancer cells. Thus, the development of novel small molecules targeting the upstream regulators, such as PKCε and cytoplasmic deacetylases that promote the nuclear translocation of HDAC6, may provide an opportunity to prevent the metastasis of lung cancer.

## MATERIALS AND METHODS

### NSCLC tissue specimens and tissue micro-array (TMA) construction

Tumor tissue specimens from 134 patients who underwent lobectomies or pneumonectomies for primary NSCLC were obtained from Kaohsiung Medical University Hospital with IRB approval (KMUH-IRB-2011-0286 and KMUH-IRB-2011-0424). The tumors were histologically examined and classified using the 2004 World Health Organization (WHO) International Classification of Lung Tumors [39]. The tumor size, local invasion, lymph node involvement, distal metastasis and final disease stage were determined according to the definition of the AJCC TNM classification of lung cancer [40]. Follow-up was up to 200 months.

### Immunohistochemistry and image processing

The detailed protocol for immunohistochemistry was as previously described [41, 42]. Briefly, the tissue microarray samples were dewaxed and rehydrated. After antigen retrieval and blocking with 3% H_2_O_2_ and 10% normal goat serum (NGS), the slides were incubated with rabbit polyclonal antibodies against HDAC6 (Santa Cruz Biotechnology, Inc., Santa Cruz, CA, USA), followed by biotin-conjugated anti-rabbit secondary antibody and polymer-HRP reagent using the ABC kit from Vector Laboratory (Burlingame, CA, USA). The peroxidase activity was visualized with diaminobenzidine tetrahydroxychloride (DAB) solution (Vector Laboratory). The sections were counterstained with hematoxylin.

HDAC6 staining intensity was quantified using the HistoQuest software (TissueGnostics, Vienna, Austria) as described previously [43]. Briefly, the tumor parts were selected as the region of interest (ROI) by the clinicians, Dr. Ming-Shyan Huang and Dr. Chih-Jen Yang. All sections were coded and analyzed by observers blinded for the clinical data. For the HistoQuest software, the colors of the DAB master marker (HDAC6) and the hematoxylin nonmaster marker (nucleus) were defined according to the positive signal in the representative section. The DAB signal intensity in the cytoplasm and nucleus was individually measured and defined as HDAC6-cytoplasm and HDAC6-nucleus. The intensity of HDAC6 staining signal was range from 0 to 255. The cytoplasmic and nuclear HDAC6-positive cells were defined using the cutoff value 150. The high and low frequency of cytoplasmic and nuclear HDAC6-positive cells in the ROI was determined using the cutoff value 50% and 20% respectively.

### Cell culture

A549 and H661 cell lines were purchased from the American Type Culture Collection. The A549 cells were cultured in Dulbecco's modified Eagle medium (DMEM) (Gibco, Grand Island, NY, USA). The H661 cells were cultured in RPMI 1640 medium. The medium was supplemented with 10% fetal bovine serum (FBS) (Hyclone, Logan, UT, USA), penicillin and streptomycin (100 U/mL), and 2 mM L-glutamine. All cells were incubated in 5% CO_2_ in a 37°C incubator. For long-term culturing, mycoplasma test was conducted every month for all the cell lines.

### Proliferation assay

Cell proliferation rate was determined by using the XTT colorimetric cell proliferation assay (Roche Diagnostics Corporation, Indianapolis, IN, USA). Briefly, the 2, 000 cells/well were seeded in 96-well plates and incubated at 37°C to allow the cell growth for 5 days. After incubation, the culture medium in the 96-well plates was removed, and 100 μl of fresh culture medium and a pre-formulated 50 ml XTT mixed reagent (XTT reagent: electronically coupled reagent = 50:1) was added. The culture plate will be incubated at 37°C for 4 hours. The absorbance of the samples was measured with a spectrophotometer at a wavelength of 475 nm.

### Migration assay

Ibidi wound healing inserts (Ibidi GmbH, Martinsried, Germany) were placed in the 6-well plates. The cells were suspended in growth media (5 × 10^5^ cells/ml) and added to each insert. The plates were incubated 37°C, 5% CO_2_ in a humidified atmosphere overnight, and then transferred into the incubation chamber equipped on the inverted microscope for time lapse imaging.

### Invasion assay

Cell invasion was performed using the Matrigel-coated film insert (8 mm pore size) fitted into 24-well invasion chambers (BD Biosciences, San Jose, CA, USA) [44]. After 24 h incubation, the filter inserts were removed from the wells and the cells on the top surface of the filter were removed using cotton swabs. The cells on the bottom surface of the filter were stained with 4′, 6-diamidino-2-phenylindole (DAPI) for 1 min, washed 3 times with PBS, and the cell number was counted under an Olympus fluorescence microscope.

### Plasmid construction and transfection

The plasmid pcDNA3.1(+)-flag-HDAC6 obtained from Addgene (Plasmid number #13823). The pcDNA3.1-ΔN-HDAC6-His plasmid was constructed by restriction enzyme digestion (NotI and EcoRI) of the full-length human HDAC6 cDNA from the plasmid pcDNA3.1(+)-flag-HDAC6 and ligated into the NotI/EcoRI restriction enzyme sites within the pcDNA3.1C-His plasmid. The pcDNA3.1(+)-flag-HDAC6 and pcDNA3.1-ΔN-HDAC6-His plasmids were transfected into A549 and H661 cells (3 × 10^6^ cells/10-cm dish) using Lipofectamine 2000 (Invitrogen, Carlsbad, CA, USA).

### Real-time PCR

The cDNA was synthesized from the total RNA samples using a Verso cDNA Synthesis Kit (Thermo Fisher Scientific, Waltham, MA, USA), followed by PCR reactions containing TaqMan^®^ Universal PCR Master Mix (Applied Biosystems Inc, Foster City, CA, USA) and the primers for HDAC6 (Forward: 5′-gatcaggccatattttatgc3′ and Reverse: 5′-gatgttcttcacatctagga-3′), MMP2 (Forward 5′-ccactgccttcgatacac-3′ and Reverse: 5′-gagccactctctggaatcttaaa-3′), and GAPDH (Forward 5′-caagggcatcctgggctac-3′ and Reverse: 5′-caccctgttgctgtagccaa-3′).

### Immunoblotting analysis

Total lyses of treated A549 and H661 cells were extracted using EBC buffer, as described previously [42]. The rabbit polyclonal anti-HDAC6 and anti-MMP2 antibodies and mouse monoclonal anti-Acetyl-α-tubulin antibody were purchased from Santa Cruz. The mouse monoclonal anti-β-actin antibody was obtained from Sigma (St. Louis, MO, USA).

### Immunofluorescence

A549 and H661 cells grown on glass slides were rinsed with PBS and fixed in 4% formaldehyde at room temperature for 20 min. The cells were washed 3 times with PBS and processed for indirect immunofluorescence as previously described [42]. The slides were incubated with the mouse anti-acetylated-α-tubulin antibody, rabbit anti-HDAC6, anti-His (1:400, Santa Cruz biotechnology, Inc.), and anti-Flag antibody (1:400, Sigma). The cells were then washed and incubated with FITC-labeled goat anti-rabbit or TR-labeled goat anti-mouse (1:400, Santa Cruz biotechnology, Inc.) antibody. Images were taken with an Olympus fluorescence microscope.

### Immunoprecipitation assay

Immunoprecipitation assay was performed according to previous report [42]. Briefly, cell lyses were prepared in the EBC buffer. Pre-cleaning was performed twice with uncoated protein G-sepharosebeads. The supernatant was further incubated with mouse monoclonal antibody for anti-Acetyl-NF-κB p65, and rabbit polyclonalantibodies for HDAC6, NF-κB, and His (Santa Cruz Biotechnology, Inc.), and rabbit polyclonal antibody for Flag (Sigma, St. Louis, MO, USA). Immune complexeswere precipitated with protein G-sepharose beads, followed by SDS-PAGE and Western blot assay.

### Chromatin immunoprecipitation (ChIP) assay

The cells were cross-linked with 1% formaldehyde at room temperature for 20 min and then washed with cold PBS. The cell pellet was re-suspended in EBC buffer, followed by sonication to the DNA length of 200–500 bp. Antibodies and protein G beads were added to each of the samples, which were then rotated at 4°C overnight. The DNA was dissolved in TE buffer and analyzed by PCR. Primers used for PCR were from MMP2 promoter sequences: Forward: 5′-gaccattccttcccgttcc-3′ and Reverse: 5′-tttccccggccgcctgc-3′.

### Statistical analysis

The comparison of cytoplasmic and nuclear HDAC6 intensity in normal tissues and tumors was performed using two-tailed Student's *t* test. Groups of patients with different subcellular HDAC6 distributions were correlated with clinicopathological characteristics using chi-square method. Survival curves were analysed using the Kaplan-Meier method, and the Cox proportional hazards regression was used to test the prognostic significance of factors in the univariate and multivariate models. All statistical tests were two-sided. *P* < 0.05 was considered significant. Analysis was performed using SPSS (Statistical Package for the Social Sciences, version 19.0) software.

## SUPPLEMENTARY FIGURES AND TABLES


