# Supplementary material for: Nuclear HDAC6 inhibits invasion by suppressing NF-κB/MMP2 and is inversely correlated with metastasis of non-small cell lung cancer
Source: Oncotarget. 2015 Sep 15;6(30):30263–76. doi: 10.18632/oncotarget.4749 (PMC4745796; doi:10.18632/oncotarget.4749)
Supplement: Supplementary file 1 [file oncotarget-06-30263-s001.pdf]

## SUPPLEMENTARY FIGURES AND TABLES

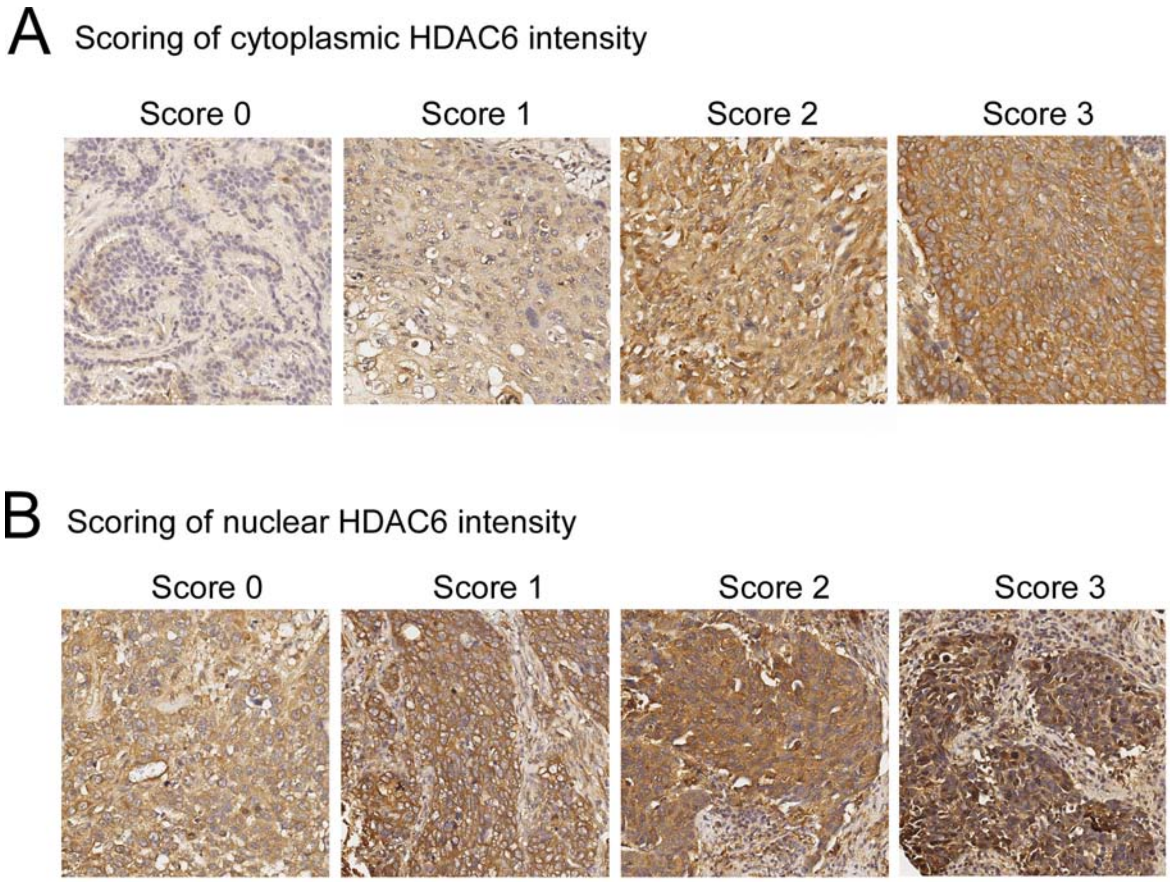


**Supplementary Figure S1: The immunohistochemistry scoring of HDAC6 expression. A.** The representative images showed the scoring of the cytoplasmic HDAC6 according to the staining intensity in the cytoplasm. **B.** The representative images showed the scoring of the nuclear HDAC6 according to the staining intensity in the nucli.

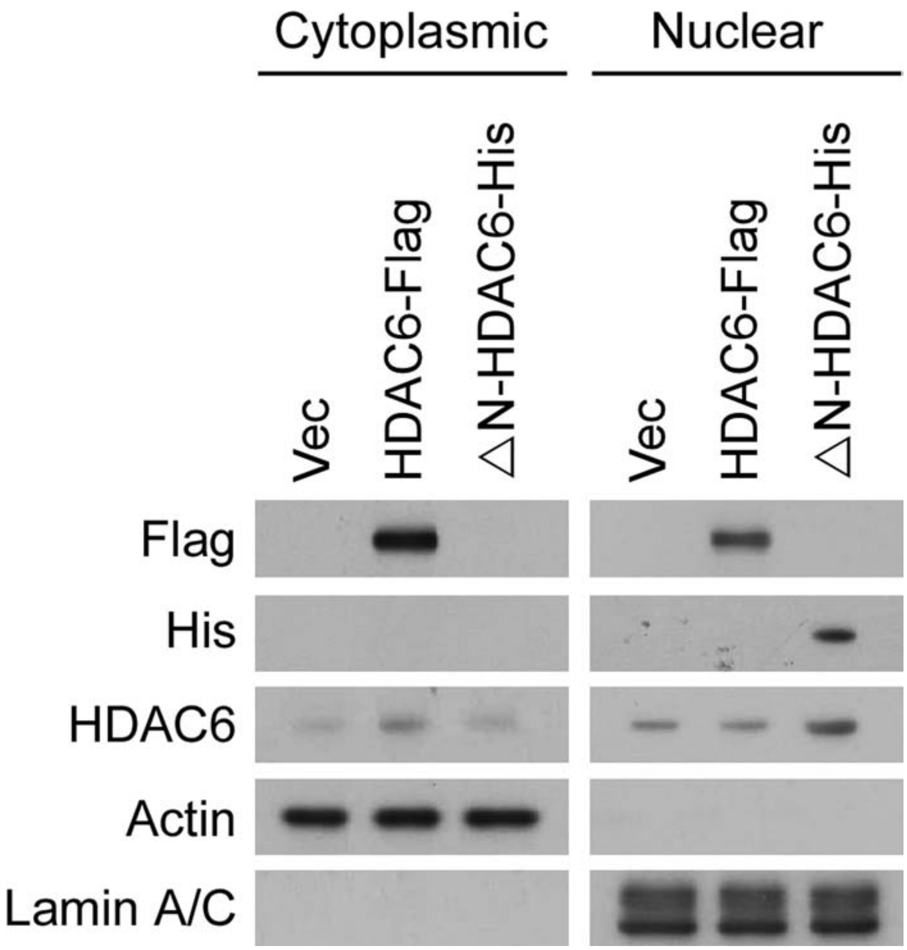


**Supplementary Figure S2: Enforced nuclear localization of ΔN-HDAC6-His in A549 cells.** The A549 cells were transiently transfected with the vector (pcDNA3.1), HDAC6-Flag and ΔN-HDAC6-His plasm ids by Lipofectamine 200 for 24 hours. The cytoplasmic and nuclear proteins were fractionated using NE-PER Nuclear and Cytoplasmic Extraction Reagents. The results from Western blot showed that the exogenous wild type HDAC6-Flag was mainly expressed in cytoplasm, although the nuclear HDAC6-Flag could also be detected. In parallel, the ΔN-HDAC6-His protein was localized in the nucleus only.

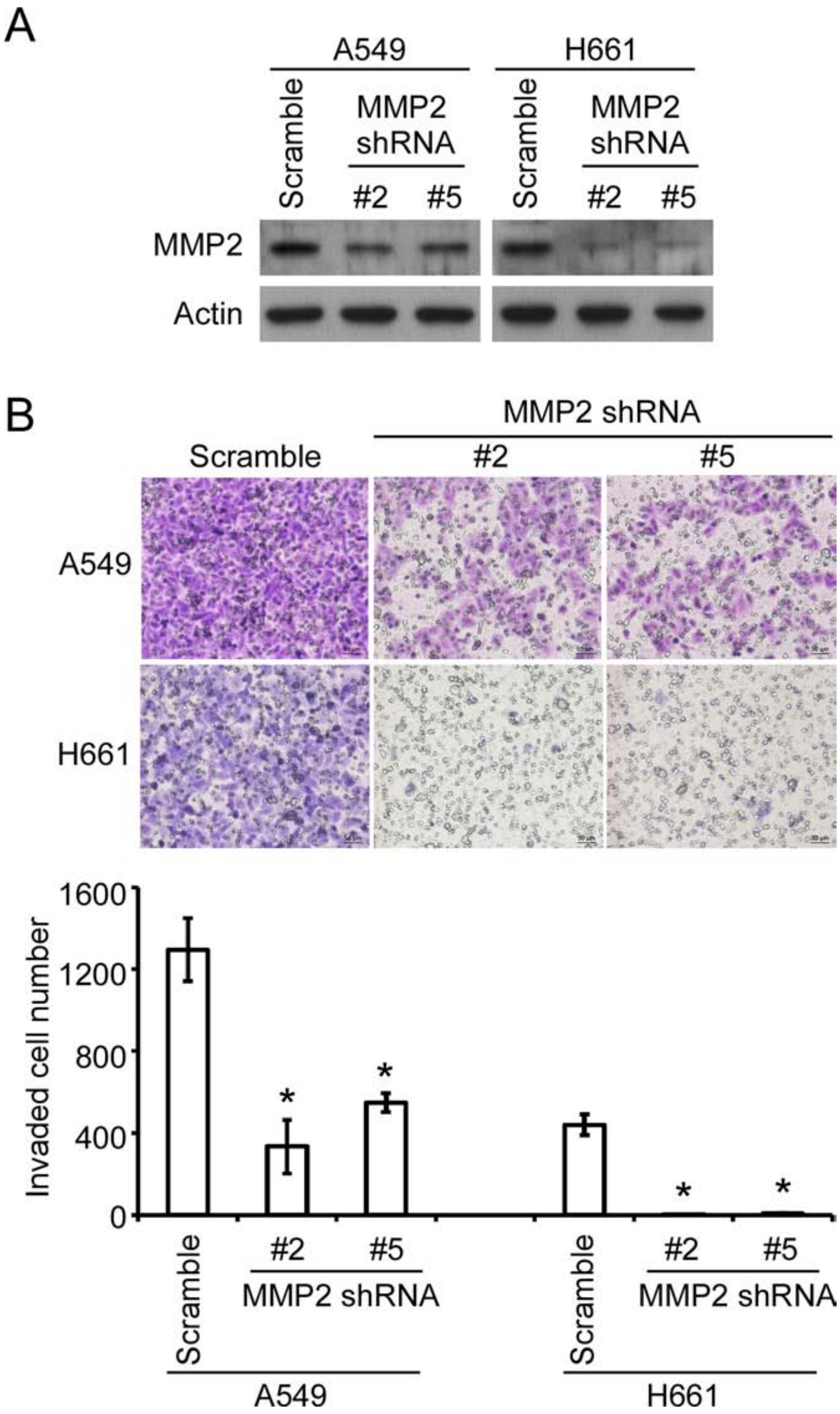


**Supplementary Figure S3: Knockdown of MMP2 reduces invasion of A549 and H661 cells.** The expression of MMP2 was knocked down using lentiviral MMP2 shRNAs (clone #2 and clone #5). After infection of the lentivirus, carrying scramble, clone #2 and clone #5 shRNAs, for 3 days, the stable clones were established by incubating the infected A549 and H661 cells in puromycin for 48 hours. **A.** The results from Western blot showed that the protein expression of MMP2 was significantly down-regulated in both lentiviral shRNA-infected cell lines. **B.** Knockdown of MMP2 significantly reduced the invasion ability of A549 and H661 cells. *$p < 0.05$

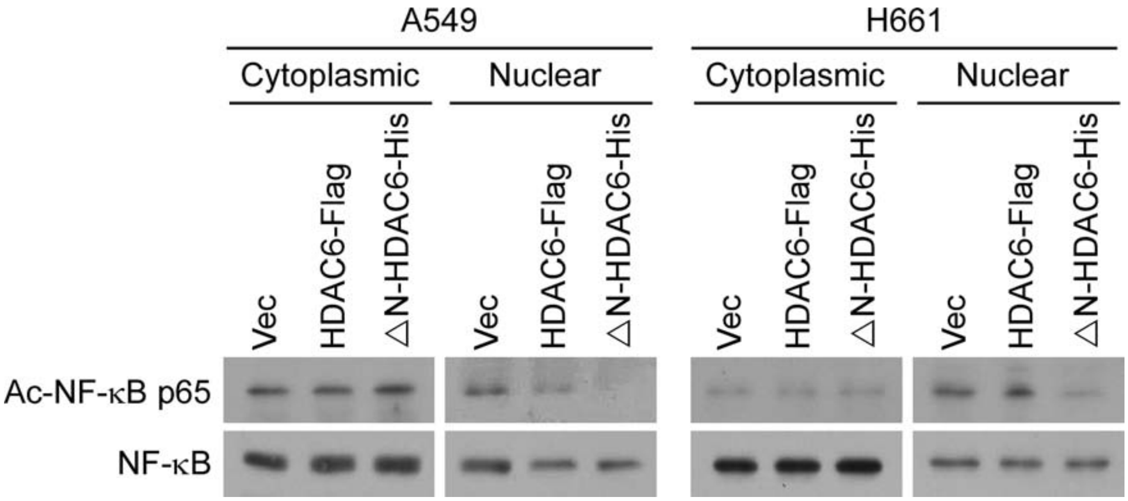


**Supplementary Figure S4: HDAC6-mediated deacetylation of NF-κB p65 subunit occurs in nucleus.** A549 cells were transfected with pcDNA3.1 and wild type (HDAC6-Flag) and NES-deleted HDAC6 (ΔN-HDAC6-His) plasmids. The Western blot was performed to analyze the acetylation of p65 subunit in the cytoplasmic and nuclear protein fractions. The results showed that ectopic expression of ΔN-HDAC6-His significantly reduced the acetylation of p65 subunit in the nuclear fractions, but not in the cytoplasmic fraction, in both A549 and H661 cell lines, suggesting that the deacetylation of p65 subunit by HDAC6 occurred in the nucli.

**Supplementary Table S1. Summary of clinicopathological features of 134 NSCLC patients with primary tumors**

| Characteristics | Number | % |
|---|---|---|
| Age(y) | | |
| ≤60 | 55 | 41 |
| >60 | 79 | 59 |
| Gender | | |
| Male | 63 | 47 |
| Female | 71 | 53 |
| Stage | | |
| I | 38 | 28 |
| II | 16 | 12 |
| III | 35 | 26 |
| IV | 45 | 34 |
| pT (primary tumor) | | |
| pT1 | 22 | 17 |
| pT2 | 70 | 52 |
| pT3 | 8 | 6 |
| pT4 | 34 | 25 |
| pN (regional lymph node metastasis) | | |
| pN0 | 50 | 37 |
| pN1–3 | 84 | 63 |
| pM (distant lymph node metastasis) | | |
| pM0 | 88 | 66 |
| pM1 | 46 | 34 |
| Histologic type | | |
| Adenocarcinoma | 81 | 60 |
| Squamous cell | 43 | 32 |
| Large cell | 10 | 8 |
| Recurrence | | |
| No | 64 | 48 |
| Yes | 70 | 52 |
| Smoking | | |
| No | 78 | 58 |
| Yes | 56 | 42 |

**Supplementary Table S2. Analysis of the frequency of cytoplasmic and nuclear HDAC6-positive cells in 134 NSCLC patients**

| | Frequency of HDAC6-positive cells (%) | |
|---|---|---|
| | Cytoplasmic | Nuclear |
| Mean | 63.73 | 31.63 |
| Std. deviation | 2.10 | 1.62 |
| Maximum | 97.38 | 72.98 |
| Minimum | 0.01 | 0.01 |
| Median | 67.64 | 28.73 |
| Percentile | | |
| 25 | 52.69 | 16.82 |
| 75 | 83.12 | 46.63 |

**Supplementary Table S3. Relationship between the frequency of HDAC6 cytoplasmic localization and clinicopathological factors in 134 NSCLC patients**

| | Cytoplasmic HADC6+ frequency | | |
|---|---|---|---|
| **Characteristics** | **Low (*n* = 44)** | **High (*n* = 90)** | **[*]*P* value** |
| Age | | | 0.104[†] |
| Years (mean ± SD) | 59.5 ± 10.8 | 62.7 ± 10.1 | |
| Gender | | | 0.534[‡] |
| Male | 25 | 46 | |
| Female | 19 | 44 | |
| Smoking status | | | 0.819[‡*] |
| No | 25 | 53 | |
| Yes | 19 | 37 | |
| Histological type | | | 0.475[‡] |
| Adenocarcinoma | 26 | 55 | |
| Squamous cell carcinoma | 13 | 30 | |
| Large cell carcinoma | 5 | 5 | |
| Stage | | | 0.071[‡] |
| I | 7 | 11 | |
| II | 11 | 25 | |
| III | 6 | 29 | |
| IV | 20 | 25 | |
| Tumor status | | | 0.918[‡] |
| T1 | 8 | 14 | |
| T2 | 22 | 48 | |
| T3 | 2 | 6 | |
| T4 | 12 | 22 | |
| Lymph node status | | | 0.704[‡] |
| N0 | 25 | 48 | |
| N1–3 | 19 | 42 | |
| Distal metastasis status | | | 0.262[‡] |
| M0 | 26 | 62 | |
| M1 | 18 | 28 | |
| Recurrence status | | | 0.272[‡] |
| No | 20 | 50 | |
| Yes | 24 | 40 | |

[*]*P* value < 0.05 was considered statistically significant (Student's *t* test for continuous variables and Pearson chi-square test for categorical variables). SD represents standard deviation. The tumor stage, tumor, lymph node, and distant metastasis status were classified according to the international system for staging lung cancer.

[†]Student's test

[‡]Chi-square test
